# A Retrospective Analysis of the Haemodynamic and Metabolic Effects of Fluid Resuscitation in Vietnamese Adults with Severe Falciparum Malaria

**DOI:** 10.1371/journal.pone.0025523

**Published:** 2011-10-11

**Authors:** Nguyen Hoan Phu, Josh Hanson, Delia Bethell, Nguyen Thi Hoang Mai, Tran Thi Hong Chau, Ly Van Chuong, Pham Phu Loc, Dinh Xuan Sinh, Arjen Dondorp, Nicholas White, Tran Tinh Hien, Nicholas Day

**Affiliations:** 1 Hospital of Tropical Diseases, Ho Chi Minh City, Vietnam; 2 Cairns Base Hospital, Cairns, Australia; 3 Worldwide Antimalarial Resistance Network, Bangkok, Thailand; 4 Mahidol-Oxford Tropical Medicine Research Unit, Faculty of Tropical Medicine, Mahidol University, Bangkok, Thailand; 5 Centre for Tropical Medicine, Nuffield Department of Clinical Medicine, University of Oxford, Oxford, United Kingdom; Bernhard Nocht Institute for Tropical Medicine, Germany

## Abstract

**Background:**

Optimising the fluid resuscitation of patients with severe malaria is a simple and potentially cost-effective intervention. Current WHO guidelines recommend central venous pressure (CVP) guided, crystalloid based, resuscitation in adults.

**Methods:**

Prospectively collected haemodynamic data from intervention trials in Vietnamese adults with severe malaria were analysed retrospectively to assess the responses to fluid resuscitation.

**Results:**

43 patients were studied of whom 24 received a fluid load. The fluid load resulted in an increase in cardiac index (mean increase: 0.75 L/min/m^2^ (95% Confidence interval (CI): 0.41 to 1.1)), but no significant change in acid-base status post resuscitation (mean increase base deficit 0.6 mmol/L (95% CI: −0.1 to 1.3). The CVP and PAoP (pulmonary artery occlusion pressure) were highly inter-correlated (r_s_ = 0.7, p<0.0001), but neither were correlated with acid-base status (arterial pH, serum bicarbonate, base deficit) or respiratory status (PaO_2_/FiO_2_ ratio). There was no correlation between the oxygen delivery (DO_2_) and base deficit at the 63 time-points where they were assessed simultaneously (r_s_ = −0.09, p = 0.46).

**Conclusions:**

In adults with severe falciparum malaria there was no observed improvement in patient outcomes or acid-base status with fluid loading. Neither CVP nor PAoP correlated with markers of end-organ perfusion or respiratory status, suggesting these measures are poor predictors of their fluid resuscitation needs.

## Introduction

The introduction of artemisinin-based therapies has revolutionised the care of patients with malaria. The mortality of severe malaria in Asian adults was reduced by 35% with parenteral artesunate treatment compared with the previous standard parenteral quinine [1]. However, even with intravenous artesunate treatment, 15 to 25% of adult patients with severe malaria will die; the majority during the first 48 hours of their hospitalisation [1]. No adjunctive chemotherapy has been shown to be of benefit in severe falciparum malaria and there are surprisingly few clinical data regarding the optimal supportive care of patients in the early stages of their hospitalisation [2], [3]. In contrast impressive improvements in mortality have been demonstrated using early goal-directed therapy in patients with sepsis [4] and, in resource poor settings, with simple fluid resuscitation protocols in dengue fever [5]. Indeed, even using quinine treatment, patients with severe malaria admitted to an excellent French intensive care unit had a mortality rate of 11% [6], suggesting that improved supportive care can lead to better outcomes in malaria as well.

Small series have confirmed that some adult patients with severe malaria are hypovolaemic, or at least have a reduced effective circulating volume [7], [8]. This hypovolaemia has the potential to exacerbate renal failure and lactic acidosis, two of the most significant contributors to mortality in severe malaria [9]. It might be expected that prompt fluid resuscitation would improve acidosis and pre-renal renal failure and therefore improve outcomes. By analogy early goal directed fluid resuscitation is a key feature of international sepsis guidelines [10]. However, non-cardiogenic pulmonary oedema is also a manifestation of severe malaria in adults and with a mortality rate of 80% in resource-poor settings [11], physicians have tended to err on the side of caution with fluid replacement.

If the patient is profoundly anaemic, transfusion of whole blood is clearly preferred [12]. However, whilst there are theoretical advantages in using colloid rather than crystalloid as a resuscitation agent [13], in a recent trial of critically ill African children – the majority of whom had severe malaria – those resuscitated aggressively with colloid and crystalloid had equally poor outcomes [14], and had a higher mortality than those managed conservatively. Furthermore while there are no data for adults with severe malaria, in a heterogeneous population of adults admitted to well equipped and well staffed intensive care units (ICU) with a range of non-malarious conditions there was no advantage in resuscitating these patients with albumin compared to crystalloid [15].

With few data to guide them, WHO guidelines emphasise the physical examination of patients; “If there is evidence (on physical examination) of dehydration, give only isotonic fluid (0.9% saline) by intravenous infusion, but avoid circulatory overload as it may rapidly precipitate fatal pulmonary oedema” [16]. Others have pointed out that, in the absence of precipitous fluid loss such as in cholera, in severe infections there is often little correlation between the physical assessment of hypovolaemia and true volume status [17]. Tacitly acknowledging this, the WHO guidelines also suggest that patients with severe malaria should, if possible, have a central line inserted and the central venous pressure (CVP) should be used to assist in the assessment of volume status [16].

To characterise the effects of fluid resuscitation in adults with severe malaria, we reviewed retrospectively the clinical and biochemical data of patients with severe malaria who were enrolled in prospective haemodynamic studies. We aimed to determine whether there was any evidence for improved outcomes when patients in these studies were resuscitated with normal saline or colloid. We also assessed the utility of central venous pressure (CVP) and pulmonary artery occlusion pressure (PAoP) as measures of volume status and thus fluid requirements in patients with severe malaria.

## Methods

### Ethics statement

Written informed consent was obtained from each patient or, in the case of comatose patients, their attendant relative. All studies were approved by the Ethical and Scientific Committee of the Hospital for Tropical Diseases. As the fluid resuscitation of patients guided by invasive pressure measurements represented standard of care at the time, consent was not specifically sought for the collection of data for this retrospective chart review.

### Patient selection

The original studies were carried out in a purpose-built intensive care unit at the Hospital for Tropical Diseases in Ho Chi Minh City, a hospital that acts as an infectious disease referral centre for much of southern Viet Nam. In 1993 a purpose built intensive care unit for the management of severe malaria was built and equipped for haemodynamic monitoring. Patients were included in this review if they had invasive haemodynamic monitoring performed as part of the initial study. They were considered to have severe malaria if they had asexual forms of *Plasmodium falciparum* on their peripheral blood smear and at least one of the following criteria of severity: acute renal failure with oliguria and plasma creatinine of >3 mg/dL (265 µmol/L); hypoglycaemia (plasma glucose, <40 mg/dL (2.2 mmol/L)); shock with systolic blood pressure of <80 mm Hg; metabolic acidosis with a base deficit of >10 mmol/L; venous plasma lactate of >4 mmol/L; or pulmonary oedema [18]. These criteria are a stricter modification of the widely used World Health Organisation criteria for severe malaria [19].

### Patient management

A full history was taken from either the patient or the attendant relatives and a detailed physical examination was performed. The patient was weighed, and body length was recorded. Baseline blood samples were taken for full haematology, quantitative parasite count, clotting studies, biochemistry (including arterial and venous gases, pH, plasma lactate and glucose), and blood cultures.

An arterial catheter was inserted into the femoral artery, and a flow-directed pulmonary artery catheter (Abbott Laboratories, North Chicago, IL) was introduced via the internal jugular route under fluoroscopy. Continuously monitored intravascular pressures (arterial pressure, CVP, PAoP), electrocardiogram, and oximetric oxygen saturation were recorded on a multifunction monitor (Hewlett-Packard, Palo Alto, CA). PAoP was measured intermittently. Cardiac output was determined by thermodilution in triplicate using 10 mL boluses of cooled 5% dextrose. Body temperature was recorded as the core temperature from the pulmonary artery catheter terminal thermistor. Simultaneous arterial and mixed venous plasma lactate and glucose concentrations were determined enzymatically using dedicated on-line analysers (Analox, London, UK). Systemic vascular resistance, oxygen delivery (DO_2_), and oxygen consumption (VO_2_) were calculated and cardiac index, systemic vascular resistance index, DO_2_ index, and VO_2_ index derived using standard formulae [20]. Preparation of the patient and recording of baseline hemodynamic and metabolic measurements took 45–120 mins and did not interfere with other aspects of clinical management.

**Figure 1:**
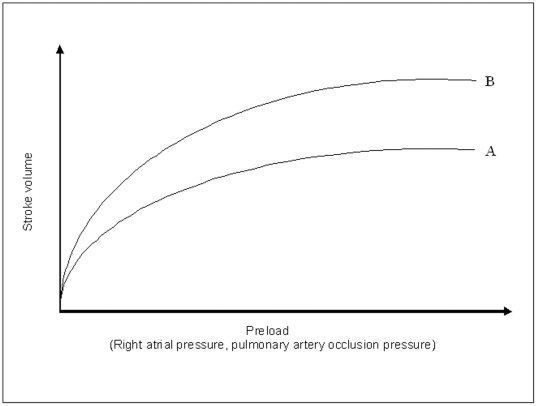
Cardiac function curve showing the relationship between preload and stroke volume (noting that cardiac output = heart rate × stroke volume). At a lower preload the stroke volume is highly sensitive to changes in preload. At a higher preload, further increases in preload lead to a reduced increase in stroke volume (the Frank-Starling mechanism). Stroke volume is also increased in the setting of increased sympathetic nervous system activation or exogenously administered inotropes. A: At rest in a person with a normal heart B: Increased sympathetic nervous system activation or exogenously administered inotropic therapy.

Depending on the study in which they were enrolled, patients received either an intramuscular quinine-loading dose regimen (20 mg/kg quinine dihydrochloride salt followed by 10 mg/kg, eight hourly), intramuscular artemether treatment (4 mg/kg stat followed by 2 mg/kg, eight hourly) (both drugs: Kunming Pharmaceutical Company, Kunming, People's Republic of China) or artesunate (Guilin Pharmaceutical Factory, Guangxi, People's Republic of China) 2.4 mg/kg iv stat, followed by 1.2 mg/kg at 12 and 24 hrs daily [18], [21]. The initial requirement for fluids was assessed clinically based on jugular venous pressure, peripheral perfusion and urine output supported subsequently by CVP and PAoP measurements. The PAoP is an indirect measure of the filling pressure of the left side of the heart and thus a gauge of volume status. According the Frank-Starling principle increasing the heart's filling pressure will lead to a greater cardiac output and hence tissue perfusion [22] (figure 1). Depending on the extent of estimated hypovolaemia and tissue hypoperfusion, patients received normal saline, a synthetic colloid (Gelofundin, B. Braun Medical, Metsungen, Germany) or no fluid loading consistent with the prevailing clinical practice of the time. Patients who received fluid loading were resuscitated to a PAoP of 9–12 mmHg. Full haemodynamic measurements including PAoP and cardiac output were made approximately every 30 mins during resuscitation, though the right atrial and arterial blood pressures were monitored continuously. In cases of shock refractory to fluid loading inotropic support with either dopamine or adrenaline was given. Oxygen was given as necessary, but the patients were not routinely mechanically ventilated as there was limited access to ventilatory support. Renal replacement was with either peritoneal dialysis or veno-venous haemofiltration.

### Statistical analysis

Results were entered into a database (Microsoft Excel, Microsoft Corp, USA) and analysed with a statistical software package (Stata 9.2, StataCorp, College Station, Texas, USA). Correlation coefficients were determined using Spearman's method. Differences between groups were analyzed using the Kruskal-Wallis and Fisher's exact tests.

## Results

**Figure 2:**
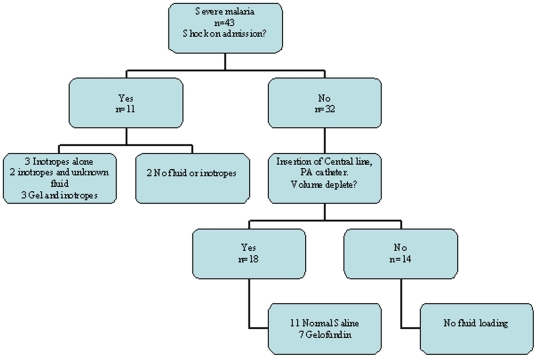
Management of the patients.

Overall there were 43 patients with severe falciparum malaria who had haemodynamic data available for analysis; 11 were admitted with haemodynamic shock, 11 had pulmonary oedema, and 30 met WHO criteria for malaria associated acute renal failure. Their management is recorded in figure 2.

The mean Glasgow Coma Scale (GCS) was 9 (95% CI: 8 to 10), the mean base deficit was 10.7 mmol/L (95% CI: 9 to 12.5) and the mean arterial lactate was 6.2 mmol/L (95% CI: 5.1 to 7.3). Fifteen patients died during their hospitalisation. On univariate analysis acid-base status on admission was a strong predictor of death: patients with a base deficit of greater than 10 mmol/L had 4 times the risk of death of those with base deficit less than 10 mmol/L (95%CI 1.01–15.86, p = 0.049). Patients with a plasma bicarbonate of less than 15 mmol/L had 6.5 times the risk of death of those with a bicarbonate of greater than 15 mmol/L (95% CI 1.4–29.7, p = 0.02). Logistic regression using purposeful selection in a model incorporating antimalarial drug used, GCS, PaO_2_/FiO_2_ ratio, plasma creatinine, total bilirubin, peripheral parasite count, age, haematocrit, cardiac index, lactate and arterial pH confirmed that acid base status, as determined by the arterial pH, was the only significant predictor of outcome.

### Fluid loading

Twenty four (56%) of the patients received a fluid load and nineteen (44%) did not. The PaO_2_/FiO_2_ ratio and the CVP and PAoP were the only variables that were statistically significantly different between these two groups (the PAoP was used to determine whether a fluid load would be given, and the CVP was highly correlated with the PAoP (r<0.0001, r_s_ = 0.75)) (table 1). The median volume of fluid administered was 918 mls (range 350–2000), and it was delivered over a median of 75 minutes (range 30 minutes to 3 hours 45 minutes). All the patients who later died completed their fluid loading.

**Table 1:** Baseline demographics of the patients, all values are means (95% CI).

Variable	Fluid load n = 24	No fluid load n = 19	P value*
Age (years)	37.7 (31.6–43.8)	35.4 (26.7–44.2)	0.39
Male (%)	21/24 (88%)	15/19(79%)	0.68<$>\raster(60%)="rg2"<$>
Parasite count (×10^3^/µl)	233 (101–365)	92.8 (36.6–149)	0.17
Glasgow Coma Score	8 (6–10)	10 (8–11)	0.15
Sodium (mmol/L)	132 (128–135)	136 (131–141)	0.16
Potassium (mmol/L)	4.1 (3.8–4.5)	4.2 (3.9–4.6)	0.63
Creatinine (µmol/L)	408 (308–508)	364 (242–486)	0.39
pH	7.33 (7.29–7.38)	7.29 (7.22–7.35)	0.12
Bicarbonate (mmol/L)	14 (11.8–16.2)	14 (11.7–16.3)	0.99
Base deficit (mmol/L)	10.3 (8–12.5)	11.3 (8.2–14.3)	0.78
Lactate (mmol/L)	6.2 (4.5–7.8)	6.2 (4.5–7.9)	0.52
Total bilirubin (µmol/L)	136 (98–173)	116 (74–160)	0.48
Haemoglobin	9.0 (7.9–10)	9.4 (7.8–10.9)	0.45
Renal failureΨ	18/24 (75%)	12/19 (63%)	0.51<$>\raster(60%)="rg2"<$>
AnuricΩ	11/24 (46%)	8/19 (42%)	1<$>\raster(60%)="rg2"<$>
Shock#	6/24 (25%)	5/19 (26%)	0.72<$>\raster(60%)="rg2"<$>
Pulmonary oedema∧	4/24 (18%)	7/19 (37%)	0.17<$>\raster(60%)="rg2"<$>
MABP (mmHg)	77.9 (71.9–83.8)	78.4 (68.2–88.7)	0.59
CVP (mmHg)	2 (1–3)	4.5 (3–6)	0.005
PAoP (mmHg)	6 (4–7)	10 (8–12)	0.001
Cardiac index (L/min/m^2^)	4.0 (3.5–4.5)	4.0 (3.4–4.6)	0.91
SVR (dyne/s/cm^−5^ m^2^)	1633 (1375–1891)	1589 (1299–1879)	0.86
Oxygen saturation (%)	96 (95–97)	95 (93–97)	0.24
PaO_2_/FiO_2_ ratio	456 (398–513)	286 (164–407)	0.02
CvO2 (%)	58.7 (54.3–62.9)	57.1 (52.2–62)	0.57
DO_2_ (mL/min/m^2^)	447 (393–502)	449 (386–512)	0.99
VO_2_ (mL/min/m^2^)	164 (151–178)	171 (148–194)	0.52
OER %	39 (34.6–42.4)	39.6 (34.6–44.6)	0.83

*p test by Kruskal Wallis, except.

^<$>\raster(60%)="rg2"<$>^Fisher's exact.

Ψ Admission serum creatinine>265 µmol/L.

Ω <50 ml urine output in first 24 hours.

# Systolic blood pressure <80 mmHg with evidence of poor end organ perfusion.

∧ Clinician's diagnosis.

MABP: Mean arterial blood pressure, PAoP: Pulmonary artery occlusion pressure, SVR: systemic vascular resistance, CvO2: mixed venous oxygen saturation. DO2: Oxygen delivery. VO2: Oxygen consumption. OER: oxygen extraction ratio.

With a fluid load there was a significant mean increase (95% CI) in Cardiac Index: 0.75 L/min/m^2^ (0.41–1.1), PAoP: 5 mmHg (3–6) CVP: 3 mmHg (1–4) and mixed venous oxygen saturation (CvO2) 4.4% (1.5–7.4), and a significant mean (95% CI) decrease in systemic vascular resistance (SVR) −242 dyne/s/cm^−5^ m^2^ (−380 to −104), oxygen consumption (VO2) −13 mL/min/m^2^(−28 to −1.8) and the oxygen extraction ratio (OER) −4.6% (−7.7 to −1.4). There was a significant decrease in haemoglobin (median, interquartile range) −0.7 g/dL (−1.6 to −0.1). Notably there was no significant change in the acid-base status of the patients or their clinical outcomes (see tables 2 and 3).

**Table 2:** Outcome by fluid load.

	Fluid load	No fluid load	p value*
Died	9/24 38%	6/19 32%	0.76
APO developed∧	1/24 4.2%	1/19 5.3%	1
CM developed∧	0/24 0%	3/19 16%	0.08
ARF developedΨ	3/24 13%	1/19 5.3%	0.62
Shock developed#	5/24 21%	6/19 32%	0.5

*p value determined by Fisher's exact. APO: Acute pulmonary oedema, CM: cerebral malaria ARF: Acute renal failure.

Ψ serum creatinine>265 µmol/L.

∧ Clinician's diagnosis.

# Systolic blood pressure <80 mmHg with evidence of poor end organ perfusion.

**Table 3:** Change in variables with fluid loading n = 24.

Variable	Mean Δ	95% CI
ΔMABP (mmHg)	2	−1 to 6
ΔCVP (mmHg)	3	1 to 4
ΔPAoP (mmHg)	5	3 to 6
Δ Cardiac index (L/min/m^2^)	0.75	0.41 to 1.1
ΔDO_2_ (mL/min/m^2^)	26	−2 to 54
Δ SVR (dyne/s/cm^−5^ m^2^)	−242	−380 to −104
ΔSaO2 (%)	0	0 to 1
ΔpO2/FiO2	−5.2	−37 to 27
ΔPaO_2_ mmHg	−4	−15 to 8
ΔPaCO_2_ mmHg	1	−1 to 2
Δ Haemoglobin (g/dL)*	−0.7	−1.6 to −0.1
Δ CvO_2_ (%)	4.4	1.5 to 7.4
ΔVO_2_ (mL/min/m^2^)	−13	−28 to −1.8
ΔO2er (%)	−4.6	−7.7 to −1.4
Δ pH	−0.01	−0.02 to 0
ΔBicarbonate (mmol/L)*	−0.1	−1 to 1
ΔBase deficit (mmol/L)	0.6	−0.1 to 1.3
ΔLactate (mmol/L)	−0.1	−0.4 to 0.6

MABP: mean arterial blood pressure, CVP: central venous pressure, PAoP: pulmonary artery occlusion pressure, SVR: systemic vascular resistance, SaO_2_: oxygen saturation, PaO_2_: partial pressure of oxygen, PaCO_2_: partial pressure of carbon dioxide, CvO_2_: mixed venous oxygen saturation, DO_2_: oxygen delivery, VO_2_: oxygen consumption, O_2_er: oxygen extraction ratio.

*Median (IQR) as non-parametric distribution.

Twenty two patients (92%) had the fluid that was used as the resuscitation agent recorded; twelve of these patients received normal saline and ten received gelofundin. The group that received gelofundin were sicker with a lower MABP, pH, and a greater base deficit and arterial lactate. Despite this, patients receiving gelofundin had a similar mortality rate to those receiving normal saline (tables 4, 5).

**Table 4:** Baseline demographics by resuscitation agent (mean (95% CI)).

Variable	Normal saline	Gelofundin	p value*
Age	37.3 (27.9–46.7)	34.3 (25 to 43.6)	0.67
Sex (male) %	12/12 (100%)	7/10 (70%)	0.08
Glasgow coma score	9 (6–11)	7 (5–9)	0.49
pH	7.39 (7.37–7.41)	7.30 (7.25–7.35)	0.004
HCO3(mmol/L)	16.6 (14.7–18.6)	11.5 (8.2–14.7)	0.009
Base deficit (mmol/L)	7.4 (5.3–9.5)	12.8 (9.2–16.4)	0.006
Lactate(arterial)(mmol/L)	4.4 (2.7–6.1)	8.1 (5–11.3)	0.03
Sodium (mmol/L)	132 (127–138)	132 (127–138)	0.84
Potassium (mmol/L)	4.4 (3.8–4.7)	3.8 (3.2–4.3)	0.07
Creatinine(µmol/L)	444 (285–602)	378 (204–551)	0.4
Total bilirubin (µmol/L)	142 (80–204)	115 (67–163)	0.64
Haemoglobin (g/dL)	9.4 (7.8–11)	8.3 (6.4–10.2)	0.11
Renal failureΨ	9/12 (75%)	8/10 (80%)	1
Anuric on admissionΩ	6/12 (50%)	4/10 (40%)	0.69
Shock on admission#	1/12 (9%)	3/10 (30%)	0.29
Cerebral malaria∧	10/12 (85%)	10/10 (100%)	0.48
APO on admission∧	2/12 (17%)	1/10 (10%)	1
MABP(mmHg)	85 (79–92)	73 (65–81)	0.009
CVP(mmHg)	2 (1–3)	1 (0–2)	0.27
PAoP(mmHg)	5 (4–6)	5 (4–7)	0.64
Cardiac index(L/min/m^2^)	4.32 (3.47–5.18)	3.87 (3.23–4.5)	0.45
CvO_2_ (%)	63.3 (59–67.6)	54 (45.9–62.1)	0.048
dO_2_ (mL/min/m^2^)	497 (427–566)	405 (308–502)	0.09
SVRdyne/s/cm^−5^ m^2^)	1707 (1275–2139)	1590 (1200–1980)	1
PaO_2_/FiO_2_ ratio	432 (341–524)	499 (403–595)	0.3
vO_2_ (mL/min/m^2^)	166 (146–186)	164 (139–191)	0.57
O_2_er(%)	35 (30–39)	43.5 (34.9–52.1)	0.06

*p by Kruskal Wallis.

Ψ Admission serum creatinine>265 µmol/L.

Ω <50 ml urine output in first 24 hours.

# Systolic blood pressure <80 mmHg with evidence of poor end organ perfusion.

∧ Clinical diagnosis.

APO: Acute pulmonary oedema, MABP: mean arterial blood pressure, MPAP: Mean pulmonary artery pressure, CVP: central venous pressure, PAoP: pulmonary artery occlusion pressure, SVR: systemic vascular resistance, SaO_2_: oxygen saturation, CvO_2_: mixed venous oxygen saturation, DO_2_: oxygen delivery, VO_2_: oxygen consumption, O_2_er: oxygen extraction ratio.

**Table 5:** Outcomes by resuscitation agent.

Variable	Normal saline	Gelofundin	p value*
Died	5/12 42%	3/10 30%	0.68
APO during hospitalisation∧	0/12 7%	1/10 10%	0.46
ARF during hospitalisationΨ	1/12 9%	2/10 20%	0.57
Shock during hospitalisation∧	3/12 25%	2/10 20%	1

*p by Fisher's exact. APO: Acute pulmonary oedema,

∧ Clinical diagnosis. ARF: Acute renal failure,

Ψ serum creatinine>265 µmol/L.

**Figure 3:**
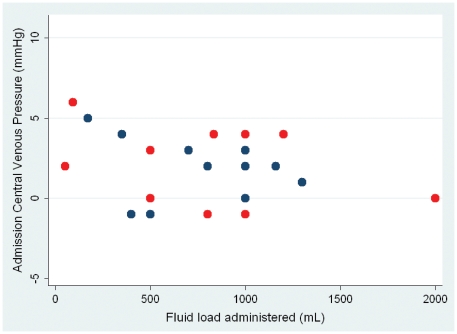
Relationship between baseline CVP and volume of fluid required to resuscitate patient (p = 0.87 r_s_ = 0.04). Blue dots survivors, red dots deaths.

Patients receiving gelofundin had a significantly greater increase in the cardiac index than those patients who received normal saline as the resuscitation fluid. Patients who received the colloid had a mean increase of 1.32 L/min/m^2^ (95% CI 0.69–1.94) as compared with a mean increase of 0.35 L/min/m^2^ (95% CI 0.1–0.61) in the patients who received normal saline (p = 0.006), although there was no difference in the volume of fluid (median (interquartile range) that the two groups received: normal saline 818 ml (500–1190), Gelofundin 1000 ml (500–1000) (p = 0.97). However, this increase in cardiac index did not translate into a statistically significant improvement in the DO_2_, CvO_2_, VO_2_, pH, base deficit or lactate (Table 6 and figure 3)

**Table 6:** Response to resuscitation agents (mean Δ (95% CI)).

Variable	Normal saline	Gelofundin	p*
ΔMABP (mmHg)	0.2 (−4.2–4.5)	6.1 (−0.9–12.3)	0.19
ΔCVP (mmHg)	2 (0–4)	5 (3–7)	0.07
ΔPAoP (mmHg)	4 (2–7)	6 (4–8)	0.43
ΔCardiac index(L/min/m^2^)	0.35 (0.1–0.61)	1.32 (0.69–1.94)	0.006
ΔDo_2_ (mL/min/m^2^)	4 (−25–32)	50 (−8–107)	0.11
ΔSVRI(dyne/s/cm^−5^ m^2^)	−154 (−253–−55)	−381(−706–−57)	0.15
ΔSaO_2_ (%)	0 (0–1)	0 (−1–1)	0.22
Δ PaO_2_/FiO_2_ ratio	25.7 (−6.8–58.2)	−38 (−106–28.4)	0.09
ΔHaemoglobin (g/dL)*	−0.7 (−1.5–−0.2)	−1.1 (−2.6–0.2)	0.37
ΔCvO_2_ (%)	2.6 (−1–6.2)	5.6 (0.12–11.2)	0.55
Δph	−0.02 (−0.04–−0.01)	0 (−0.03 −0 .02)	0.08
ΔHCO_3_ (mmol/L)	−0.4 (−1.2–0.5)	−0.2 (−1.1–0.8)	0.78
Δbase deficit	−1 (−1.8–0.1)	−0.3 (−1.6–0.9)	0.46
ΔLactate (mmol/L)	0.2 (−0.6–1)	−0.1 (−1–0.8)	0.7
ΔVo_2_ (mL/min/m^2^)	−12 (−33–10)	−8 (−33–17)	0.74
ΔO2er (%)	−2.3 (−6–1.3)	−6 (−11.8–0.2)	0.32

*p by Kruskal Wallis. MABP: mean arterial blood pressure, CVP: central venous pressure, PAoP: pulmonary artery occlusion pressure, SVRI: systemic vascular resistance index, SaO2: oxygen saturation, CvO2: mixed venous oxygen saturation, DO2: oxygen delivery, VO2: oxygen consumption, O2ER: oxygen extraction ratio.

There was no relationship between the volume of fluid administered and the patient's outcome or the patient's likelihood of developing shock, renal failure or pulmonary oedema. There was a significant relationship between the volume of fluid infused and the changes in the cardiac index (p = 0.04, r_s_ = 0.41), fall in haemoglobin (p = 0.03, r_s_ = −0.44), and the SVR (p<0.001, r_s_ = −0.63). However, there was no relationship between the volume of fluid infused and the changes in acid - base status (ΔBD p = 0.15 r_s_ = −0.37, Δlactate p = 0.39 r_s_ = −0.21, ΔHCO_3_ p = 0.19 r_s_ = −0.34, ΔpH p = 0.32 r_s_ = −0.23).

### Haemodynamic variables and acid-base status

There was no relationship between the CVP and PAoP on admission and cardiac output, oxygen delivery or acid/base status. After volume loading there was still no relationship between CVP and PAoP and these measures. The increase in CVP and PAoP with resuscitation was correlated with the increase in cardiac index, but there was no association between the change in CVP and PAoP and the changes in any other markers (see table 7).

**Table 7:** Correlation between ΔCVP and ΔPAoP.

Variable	CVPΔ	Rho*	PAoPΔ	Rho*
LactateΔ	0.56	−0.13	0.71	−0.08
BDΔ	0.88	0.03	0.62	0.13
HCO3Δ	0.59	−0.14	0.85	−0.05
CIΔ	0.06	0.39	0.44	0.16
DO_2_Δ	0.98	0.01	0.74	0.07
VO_2_Δ	0.8	0.05	0.66	0.1
O_2_erΔ	0.69	−0.08	0.66	0.1
CvO_2_Δ	0.58	0.12	0.76	−0.07
MABPΔ	0.13	0.32	0.5	0.14

*Spearman's rho, p value by Spearman's.

CVP: Central venous pressure, PAoP: Pulmonary artery occlusion pressure, BD: base deficit, HCO3: Bicarbonate. CI: cardiac index, DO_2_: Oxygen delivery, VO_2_: oxygen consumption, O_2_er: oxygen extraction ratio, CvO_2_: central mixed venous oxygen saturation, MABP: mean arterial blood pressure.

### Pressures and fluid volume requirements

**Figure 4:**
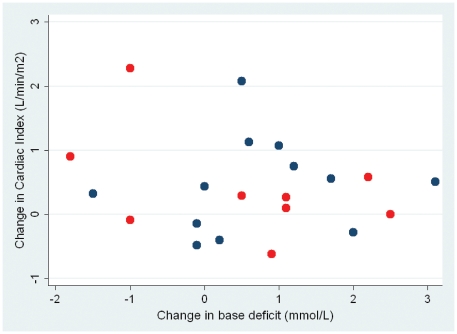
Relationship between change in base deficit against change in cardiac index with fluid loading (p = 0.95 r_s_ = 0.01). Blue dots survivors, red dots deaths.

A PAoP of 9–12 was used as the target for the resuscitation in those patients that received a fluid load. The baseline PAoP was correlated with the volume of fluid that was required to achieve this endpoint (p = 0.001, r_s_ = −0.57). However there was a great deal of overlap in these values, for instance in the 5 patients with a baseline PAoP of 4 mmHg, between 800 ml and 2000 ml of fluid was required to raise the PAoP to the target range. There was no relationship between the baseline CVP and the volume of fluid that was required to resuscitate the patient (see figure 4).

There was no significant difference between the CVP of the patients who did and did not have pulmonary oedema on admission. The median CVP (95% CI) of patients both with (n = 11) and without pulmonary oedema (n = 32) was 3 mmHg 95% (CI: 0.7–4.2 and 2–4 respectively, p = 0.89 for a difference). Similarly there was no statistical difference in the PAoP of the two groups: the median PAoP (95% CI) was 9 mmHg (4–11) in patients with pulmonary oedema and 7 mmHg (6–8) in those without the complication (p = 0.71 for a difference). There were only two patients who subsequently developed pulmonary oedema during their hospitalisation. Examination of the 56 time points in all patients for which there were simultaneous CVP, PAoP and PaO_2_/FiO_2_ measurements, revealed no relationship between either CVP (p = 0.55) or PAoP (p = 0.77) and gas exchange.

When the simultaneous assessments of macrovascular function and acid base status of all patients were pooled, there were 76 time points for which data were available. There was no relationship between the oxygen delivery and the prognostic markers of pH, bicarbonate or base deficit. There was a correlation between the mean arterial blood pressure (MABP) and pH (p = 0.03, r_s_ = 0.27), bicarbonate (p = 0.01, r_s_ = 0.34), and base deficit (p = 0.02, r_s_ = 0.31), although if the 8 data-points where the MABP was less than 60 were removed, there was no longer a correlation: pH (p = 0.7, r_s_ = 0.05), bicarbonate (p = 0.31, r_s_ = 0.15), and base deficit (p = 0.46, r_s_ = 0.11).

## Discussion

There has been considerable discussion as to the optimum fluid management of severe malaria. This small series was not powered to detect outcome differences between the treatment groups, but it provides important information on the haemodynamic and metabolic consequences of fluid administration to adults with severe malaria. There was no evidence that fluid loading improved the underlying pathological processes in severe malaria.

The rationale for fluid loading would be to improve microvascular perfusion and thus reduce the acidosis which has consistently been shown to be the strongest predictor of outcome in severe malaria [9], [23]. Once again in our series, acid base status was the strongest predictor of death. However, in the 21 patients who had a base deficit and bicarbonate measured before and after their fluid loading, the median values of these variables actually worsened.

Patients who received a fluid load did have a rise in their cardiac index, and most had a rise in their oxygen delivery, so how do we explain the lack of improvement in their acidosis? The mechanical obstruction of the microvasculature is central to the pathophysiology of severe malaria, and is a direct cause of acidosis [24], [25], [26]. Whilst fluid loading improves macrovascular status, it does little to overcome the synchronous microvascular sequestration. When data were pooled in this series there was no statistical correlation between cardiac index, oxygen delivery and the markers of acidosis.

These systemic metabolic data parallel the findings of our investigations of renal blood flow and metabolism in severe malaria. Pharmacological increases in renal blood flow with dopamine were not associated with improvements in renal function [27].

Perhaps the patients were given inadequate volumes of resuscitation fluid in this study? It would seem unlikely. After fluid loading the cardiac index improved a median of 15% (95% CI 5.4–21.5) and nearly 90% of patients had a normal cardiac index. Even if the analysis is limited to the patients who had a low DO_2_ before resuscitation the median change in BD was 0 (95% CI -1–2.5), suggesting that fluid loading is not improving the impaired tissue perfusion contributing to pathology and to outcome.

Another possible explanation for the lack of improvement in acid-base status is the possibility that any improvement in the acidosis resulting from hypoperfusion has been offset by a hyperchloraemic metabolic acidosis induced by resuscitation with chloride rich saline and Gelofundin. In recent years, use of the physicochemical Stewart approach to acid base disturbance [28] has increased the awareness that resuscitation with fluid containing supraphysiological chloride concentrations, can lead to metabolic acidosis through a reduction in the strong ion difference of plasma. Although the hyperchloraemic acidosis associated with resuscitation has not been conclusively linked to deleterious sequelae, it has been implicated in neurological dysfunction, coagulopathy, cardiac dysfunction, renal dysfunction and haemolysis [29]. Unfortunately in this series we lack sufficient data to make an assessment of the acidosis using the Stewart approach to elucidate this relationship further.

Another concern with fluid loading is that it may be deleterious in itself. It does not overcome the mechanical obstruction in the microcirculation, but with the increased pulmonary capillary permeability present in severe malaria [11], [30], it may increase the risk of pulmonary oedema - a complication with a dismal prognosis in the resource poor setting. However in this small series, only two patients developed pulmonary oedema - one of whom received a fluid challenge and the other not - precluding any meaningful analysis.

A recent large study in African children with severe malaria given fluid boluses of both saline and colloid solutions in a resource poor setting provided clear evidence of harm with vigorous fluid loading [14]. Children with severe malaria given a fluid load of 20–40 ml/kg had a relative risk of death of 1.59 when compared to patients given maintenance fluids. While there are some differences between the clinical presentation of adults and children severe malaria, the fundamental pathophysiology of the disease is similar. Our findings, when added to the findings in the African children, would certainly support caution in fluid resuscitation and would appear to add weight to the old malariologist's adage to “run them dry”.

Conversely, aggressive fluid rehydration has been shown to be highly effective in the management of Dengue haemorrhagic fever in resource poor settings [5] and is a key tenet of the Surviving Sepsis Guidelines for the management of patients with sepsis in well resourced health settings [10]. In the study of African children, those with severe malnutrition, gastroenteritis, non-infectious causes of shock were excluded from randomisation in the above study [14]. Early goal directed fluid resuscitation must still be considered the standard of care in these populations. It seems likely that when it comes to fluid resuscitation, there is no one size that fits all.

The decision to fluid load patients, and the volume of fluid that was administered, was on the basis of the PAoP, a parameter strongly linked to the CVP. Since this study was performed, the weight of clinical opinion has turned against a reliance on pressure-based measures of preload to assess the patient's volume status. Neither CVP nor PAoP are good predictors of fluid responsiveness in either healthy volunteers [31] or hypovolaemic critically ill patients [32], and current opinion in the developed world is that neither of these indices should be used to define the state of ventricular filling or the potential of patients to respond to a fluid challenge [33].

In this small series, there was no correlation between point measures of CVP and PAoP and cardiac index, oxygen delivery or acid base status, before or after fluid resuscitation. In addition to the observations about the potential limitations of CVP and PAoP being used as targets for resuscitation, there was no relationship between the CVP and PAoP and the presence of pulmonary oedema. There was also no relationship between the CVP/PAoP and the PaO_2_/FiO_2_ ratio. This accords with previous studies that have identified little correlation between pulmonary oedema and CVP [34] or PAoP [30] and is consistent with the hypothesis that pulmonary oedema in severe malaria is predominantly the result of increased pulmonary capillary permeability.

Whilst central lines offer secure access and a conduit for the administration of veno-irritant drugs, they are relatively expensive and may be associated with serious complications during insertion and maintenance [35]. In view of the limited benefit that they appear to provide the clinician in managing the patient with severe malaria in this and other series, the WHO recommendation to use them should perhaps be attenuated.

Analysing the effect of the type of fluid administered is difficult, as at baseline the patients who received gelofundin were sicker than the patients who received saline. They had a lower pH, MABP, base deficit, a higher lactate, and would be expected to have a higher mortality rate; but there was no significant difference in mortality. The patients who received gelofundin had a significantly greater increase in their cardiac index and a trend to an increased oxygen delivery, although there were no changes in base deficit and bicarbonate.

There are several flaws in our retrospective study that prevent the generation of strong recommendations based on its findings. The first and most obvious is that the small sample size limits the power of the study to detect subtle differences between the various groups. The patients were managed according to clinical judgement rather than via a defined protocol. Although renal failure is a common complication of severe malaria, the median serum creatinine in this series of 309 µmol/L (95% CI 226–414) is much higher than in other series; indeed 44% of the patients were anuric on admission. Patients with renal impairment might be expected to handle a fluid load poorly and thus our observation that these patients had little benefit from fluid loading, may reflect their renal impairment rather than any haemodynamic manifestations of their severe malaria. The number of patients with severe renal dysfunction also complicates interpretation of the acid-base data. Most patients in the series received quinine or intramuscular artemether as their anti-malarial drug treatment – both therapies have now been superseded by intravenous artesunate - and thus the mortality in the series is higher than would be expected if the patients were managed with artesunate. Finally, proponents of aggressive fluid resuscitation emphasize the importance of early treatment. While patients presenting in shock, or established organ failure is representative of the real world where they will be managed, it may be that in our study by the time resuscitation was commenced, the damage had been done.

Despite these caveats there are several observations worth making. The first is that while almost all adults with severe malaria will require intravenous fluids, the results of this study do not predict significant improvements in acid base status and outcomes as a result of this fluid resuscitation. The role of fluid resuscitation remains unclear, and although many will have true or at least effective hypovolaemia on admission, a more conservative fluid regimen may turn out to be preferable, as has been demonstrated in other patients with ARDS and septic shock [36] and was the unexpected outcome of the recent large fluids trial in African children with malaria [14].

Despite malaria claiming the lives of thousands of adults every year, relatively little is known about the optimal supportive therapies for these patients. The vast majority of patients with severe malaria will be managed in a resource poor setting, often by relatively inexperienced health care providers, with limited intensive care support. Resuscitation protocols will need to be tailored to this environment to provide appropriately easy to implement, practical guidelines for these practitioners. However the findings of this and other studies [14], [24], [25] suggest that while aggressive fluid resuscitation is relatively cheap and easier to administer in patients with severe malaria, it may be that the search for adjuvant therapy would be more fruitfully conducted at the microvascular level.
